# Interacting Virus Abundance and Transmission Intensity Underlie Tomato Spotted Wilt Virus Incidence: An Example Weather-Based Model for Cultivated Tobacco

**DOI:** 10.1371/journal.pone.0073321

**Published:** 2013-08-19

**Authors:** Thomas M. Chappell, Amanda L. P. Beaudoin, George G. Kennedy

**Affiliations:** Department of Entomology, College of Agriculture and Life Sciences, North Carolina State University, Raleigh, North Carolina, United States of America; DOE Pacific Northwest National Laboratory, United States of America

## Abstract

Through a modeling approach, we investigated weather factors that affect the summer incidence of *Tomato spotted wilt virus* (TSWV), a virus vectored exclusively by thrips, in cultivated tobacco. Aspects of thrips and plant biology that affect disease spread were treated as functions of weather, leading to a model of disease incidence informed by thrips and plant biology, and dependent on weather input variables. We found that disease incidence during the summer was influenced by weather affecting thrips activity during the preceding year, especially during a time when thrips transmit TSWV to and from the plant hosts that constitute the virus’ natural reservoir. We identified an interaction between spring precipitation and earlier weather affecting thrips, relating this to virus abundance and transmission intensity as interacting factors affecting disease incidence. Throughout, weather is the basic driver of epidemiology in the system, and our findings allowed us to detect associations between atypically high- or low-incidence years and the local climatic deviations from normal weather patterns, brought about by El Niño Southern Oscillation transitions.

## Introduction

Most viruses of plants are vectored by arthropods, and most arthropod vectors are insects [1]. Though there is variation in the transmission processes associated with different insect-vectored viral diseases of plants, these diseases have in common two important characteristics: transmission dynamics depend on vector population dynamics [2], and aggregate disease incidence depends on both the presence of virus and the process of transmission. Accordingly, models of plant viral disease vary in how they account for the role of vectors in epidemiological processes and results. For example, vector numbers [3], vector species diversity [4], and weather factors affecting vector population dynamics [5,6] have been used in models to account for vector effects on disease epidemiology of plant viruses. A thorough understanding of insect-vectored disease requires knowledge of the effects of insect population processes on disease processes. When disease-management intervention is based on epidemiological models, this knowledge of insect-mediated effects becomes critical to the design and efficacy of the intervention.

Modeling work has contributed appreciably to our understanding of how characteristics of viruses and host backgrounds affect epidemiology in viral diseases of plants. The aspects of vector dynamics that are important to transmission vary between disease systems, and vary between disease transmission mechanisms [7,8]. When a ubiquitous insect transmits virus between perennial plants, the abundance of insects potentially acting as vectors is likely to relate vector dynamics to transmission [3]. However, for a virus affecting annual plants of regimented age structure (the case in most agroecosystems), temporal dynamics of insect population affect transmission, as this transmission depends on the co-occurrence of susceptible hosts, virus, and vectoring activity on the part of insects [9]. Different models address these aspects through various approaches and offer advantages and shortcomings depending on the modeling goal. For some time, simulation models have been able to describe plant viral disease epidemics using the readily available input of weather [10], though such models’ application is limited to the instances (often grouped geographically) for which parameter values accurately relate the disease to applicable weather [11]. Analytical approaches linking metrics of vector populations to disease [12] allow for assessment of these metrics’ effects on epidemics, which in turn provides the necessary basis for simulation modeling [3]. Analytical models allow investigation of specific factors’ effects on outcomes, and facilitate further research to focus on those factors that are found to be important.

Where there is an adequate understanding of a disease system, the advantages of these different modeling approaches may to some extent be combined for the purpose of analyzing that system. *Tomato spotted wilt virus* (TSWV) in cultivated tobacco, a vector-borne virus transmitted exclusively by thrips, is one such disease system that is relatively well-characterized. Most importantly, the thrips biology related to vectoring has been studied in detail: weather effects on thrips abundance are characterized, and the relationship between weather conditions and thrips activity has been studied [5,6]. A relationship between boreal weather and TSWV-induced crop loss has also been studied [13]. TSWV is described by a history of disease management research that has resulted in the development of transmission-mitigating practices, the efficacy of which stand to benefit from improvements in understanding transmission dynamics, and the applications of which extend to other agricultural systems [14–18]. TSWV has caused crop loss in field and vegetable crops throughout the southeastern United States yearly since the first observations of TSWV in 1988, e.g. [19–22], but with appreciable variation in loss between years and locations. Pesticide application is the primary means of preventing the spread of TSWV into cultivated tobacco [23,24], and the negative effects of pesticides on crop plants means that optimizing application of pesticides depends on optimal understanding of disease risk. For TSWV in tobacco, the effectiveness of pesticide use for disease prevention depends critically on the timing of application relative to the timing of vector movement and virus transmission [23–25].

In this system, the tobacco thrips ( *Frankliniella* *fusca* Hinds) is the linkage between virus presence in reservoir hosts, and disease incidence in cultivated tobacco. Weather is expected to affect reservoir hosts and thrips vectors in different ways. For instance, precipitation should have positive effects on the growth of reservoir host plants, but it leads to mortality of juvenile thrips and suppresses flight activity of adult thrips [6,26]. Our primary goal was to better understand TSWV dynamics in this system, through studying weather’s effects on virus abundance and transmission intensity. We described these effects in terms of weather, as part of a model of disease incidence. Both virus and transmission are required for disease to arise in a host population, and models that are not adequately sensitive to one or the other of these effects can lead to expectations for incidence that are not realized. For example, two years in the history of TSWV incidence in North Carolina illustrate the relationship between virus abundance and transmission intensity. This example informs the hypotheses that virus abundance is mediated in part by winter annual plant abundance, and that transmission intensity (a composite of transmission efficiency and the frequency of events that can lead to transmission) is mediated by thrips activity. In 2002, TSWV incidence in several crop systems was unusually high [27], and the cool-season climate following cropping in that year was unusually warm with abundant precipitation. A mild and wet cool-season climate results in a landscape where winter annual plants are abundant and persist well into spring, constituting an increased TSWV reservoir and therefore increased virus abundance in that landscape. Based on the observation of unusually high virus abundance in 2002, a high disease incidence during the coming 2003 cropping season was expected; however, spring conditions in 2003 were unusually cool and wet. This resulted in decreased thrips activity during that time, and the expectation of high disease incidence in 2003 was not realized. Indeed, 2003 was a year of unusually low TSWV incidence. Studies of El Niño/La Niña Southern Oscillation (ENSO) effects during this time are consistent with the observation that climate in the southeastern United States was unusual [28], with high precipitation amounts during the spring of 2003.

The present study was undertaken to better understand factors affecting epidemiology of TSWV, using cultivated tobacco as an example. We hypothesized that a description of virus abundance in the landscape, which serves as the source for spread by tobacco thrips to tobacco, can be related to weather prior to the major dispersal peak of thrips from virus infected winter host plants in spring, and that this would interact strongly with a numerical description of transmission intensity by tobacco thrips during the current year. We were particularly interested in the time during which weather variation may most strongly affect virus abundance in the landscape before tobacco is present, possibly as recent as the current spring, or long ago as the preceding spring.

## Materials and Methods

### Ethics statement

Necessary permission was obtained for access to all fields in the described studies. Disease incidence surveys took place in agricultural plots, and permission was given by landowners for each survey to take place. No endangered or protected species were affected by the described field studies.

### Study system

**Figure 1:**
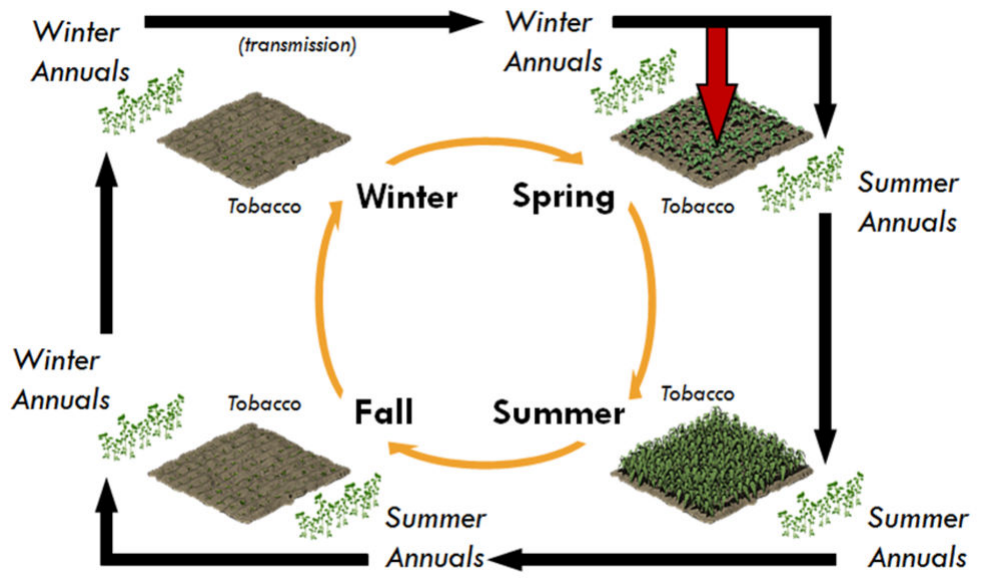
Simplified schematic of the disease system’s annual cycle, showing directionality of transmission events in time. Black arrows indicate transmission events that result in TSWV’s continuous presence in the landscape. The single red arrow indicates transmission from winter annuals to tobacco in early spring; note that transmission from tobacco to other plants does not occur to influence the cycle. Tobacco is shown to be present in spring and summer, and absent during fall and winter.

**Figure 2:**
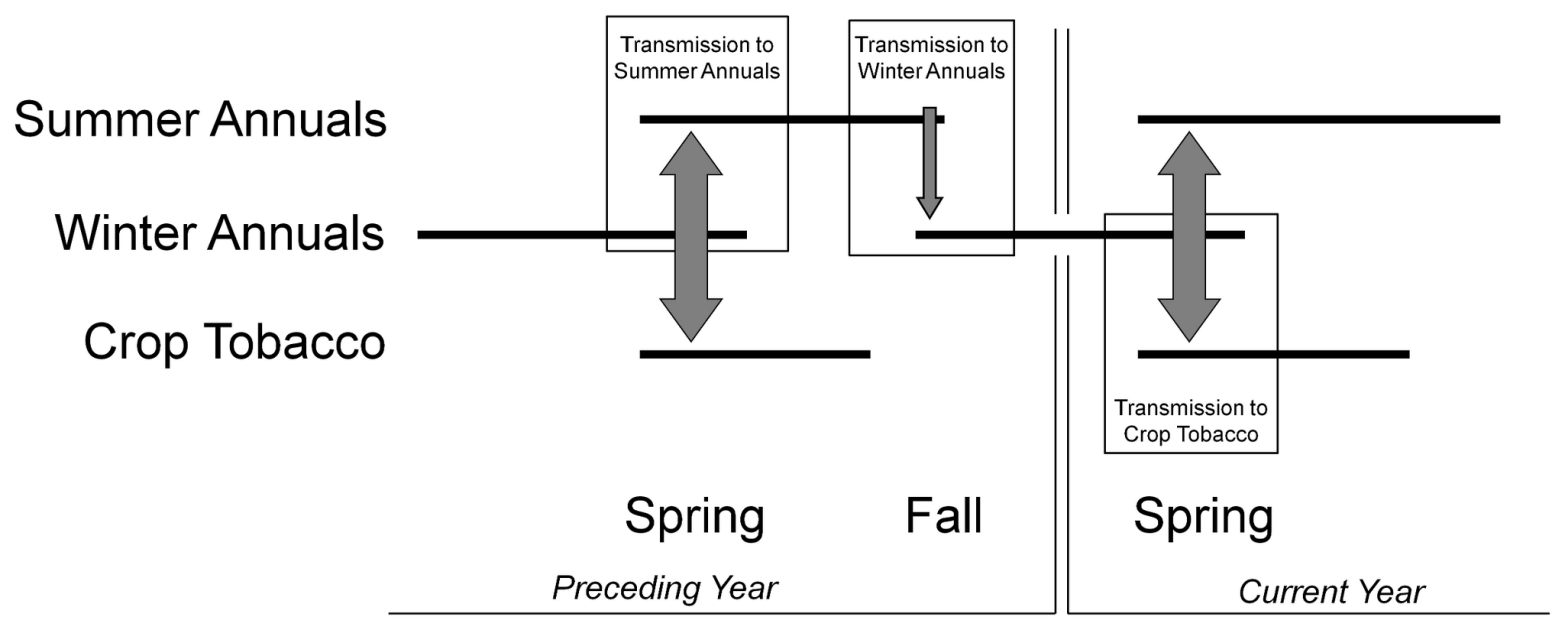
Transmission event timeline showing the occurrence of different host groups (summer and winter annual weeds, and crop tobacco) in time, and an interpretation of how and when relevant weather parameters influence the disease system. Note crops in TSWV susceptible crop hosts in the tobacco production regions are harvested and removed from the agroecosystem before winter annuals germinate.

TSWV is a *Tospovirus* vectored by thrips. In tobacco grown in North Carolina, the tobacco thrips, *Frankliniella* *fusca* , is the primary vector [29]. During the course of a year, TSWV and *F* *. fusca* spread through a sequence of weed and crop hosts [29–33]. Winter annual weeds serve as overwintering hosts of *F* *. fusca* and TSWV, and are the principal sources for spread of TSWV into crops in spring (Figure 1). Subsequent spread of TSWV among winter weeds in late winter and early spring by *F* *. fusca* results in an increase in the abundance of infected plants that serve as sources for spread of TSWV into susceptible crops and summer weed hosts in spring [5,31,32]. In the fall, TSWV is spread from summer hosts to winter weed hosts by thrips dispersing from infected summer hosts when they senesce in the fall (Figure 2). *F* *. fusca* populations decline in late June or early July and remain very low throughout the remainder of the year. Hence there is a very limited spread of TSWV among weed hosts during the summer. Crops grown in the tobacco production areas of North Carolina that become infected by TSWV in spring are harvested in mid to late summer, well before *F* *. fusca* disperse from summer weeds to their winter weed hosts. Primary spread of TSWV from non-crop hosts to susceptible crops by *F* *. fusca* in spring accounts for essentially all TSWV infection in tobacco, which does not support reproducing populations of *F* *. fusca* *.*

Size and development of late winter and spring populations of *F* *. fusca* , which account for the majority of the early season spread of TSWV, is determined largely by temperature, rainfall and host plant suitability [5]. *F* *. fusca* eggs hatch after a few days, the length of required time varying with temperature. The development of egg to adult requires 237 degree-days at a lower developmental threshold of 10.5° Celsius [34], typically taking three to four weeks during the late spring and early summer when tobacco is grown. Thrips become vectors of TSWV only if they acquire the virus early in the larval stage of development; adult thrips do not become viruliferous upon feeding on infected plant tissue [35]. The developmental window for conversion of a juvenile thrips to a vector is narrow, and the relationship between thrips development and temperature is straightforward, so that modeling thrips generational turnover and estimating peak transmission activity is also relatively straightforward. Other aspects of the disease system in North Carolina are more probabilistic, such as the relationship between winter and spring weather variables’ effects on plants *vs.* thrips. These variables affect the abundance of annual weeds that serve as reservoir hosts to TSWV [20], and affect the development and spread of TSWV by *F* *. fusca* populations overwintering on these hosts. Epidemics in tobacco can result when two conditions are met: TSWV is abundant in host plants near tobacco fields at rates conducive to epidemic spread, and tobacco thrips are abundant and active, transmitting TSWV into tobacco at an epidemic rate.

### Data

#### TSWV Incidence.

The North Carolina Cooperative Extension Service (NCCES) receives reports annually from county extension agents that include end-of-season estimates of TSWV incidence in tobacco at the county level. The reported incidence in these data is at the level of one datum per county per year, and exists for most counties in which tobacco is grown. Reports include counties where tobacco is grown but TSWV is rare or apparently absent, and counties where TSWV incidence in tobacco is relatively high during most years. TSWV incidence is reported as the proportion of diseased plants among the total tobacco in that county at the end of the season, and the report represents that agent’s assessment of the rate of TSWV infection during the given year. Additionally, we surveyed tobacco fields in several counties to assess end-of-season disease incidence, visiting tobacco fields at the end of the growing season and observing 300-500 plants per field for symptoms of TSWV infection. These data are of greater within-county replication (between two and four fields per county were surveyed) but describe fewer counties than do the statewide NCCES reports: they are representative samples instead of representative summaries as are the NCCES reports. County-wise, yearly incidence reports from the NCCES were available for 116 county-years, distributed over 29 counties and 8 years. Our surveys of fields for representative incidence observations cover 27 county-years, including data from 14 counties over 3 years. The NCCES reports also include data on the usage of the pesticide imidacloprid, which has been demonstrated in small-plot studies to decrease TSWV incidence in crops [36–39].

To confirm that our field surveys and the NCCES reports could be reasonably pooled and analyzed together, a paired sample t-test was conducted to compare incidence measures where survey and NCCES observations both exist for a given county-year. A finding of no significant difference between paired observations (one from the NCCES data, and one from our surveys of incidence) where a record from each dataset exists for one county would indicate that sampling methods were not inconsistent.

#### Climate

At the time of this study, the North Carolina State Climate Office’s (SCO) Climate Retrieval and Observations Network Of the Southeast (CRONOS) database included records from 1,524 weather sites in North Carolina. Thirty-two of these sites are members of the SCO’s ECONET monitoring and reporting network, and are administered to be the most consistent and reliable of the sites composing the CRONOS network. ECONET sites are distributed over 25 counties in North Carolina, and the intent for the ECONET network is for eventual coverage of each county in the state.

Weather data used for model development in this study were assembled primarily from ECONET weather sites and from other CRONOS sites where an ECONET site did not exist in or very near a county for which disease incidence data were reported. For each county and year in which there was such an incidence report, weather data consisting of average daily air temperature and the sum of daily precipitation were assembled to describe the weather of a county from September 1 preceding the TSWV-incidence report year through June 1 of the report year.

#### Dispersing thrips estimation

Morsello and Kennedy [6] studied the effects of temperature and precipitation on *F* *. fusca* dispersal, and we use the methods of these authors to compute estimates of thrips dispersal for use in modeling TSWV incidence. A suite of regression models was developed to describe thrips dispersal during each of four discrete two-week intervals during spring, with regression equations varying between intervals to describe differences in thrips dispersal patterns as the spring season progresses [6]. Input variables in these models included cumulative thrips developmental degree days with a base temperature of 10.5 °C, days with temperature above 20 °C (the threshold above which the temperature is favorable for thrips flight), and a treatment of rainfall to include cumulative volume of precipitation and days during which rainfall occurred [6]. We use this suite of models to represent transmission intensity through an estimate of cumulative thrips dispersal (the sum of predictions from each of the four time periods, where dispersal is measured in units of adult thrips caught on sticky traps), with one change to the methods of Morsello and Kennedy [6]. Because these models were developed using insect trap data mostly from the coastal plain of North Carolina, whereas we attempt to model disease incidence more broadly across the state, we have adjusted the date at which degree day accumulation begins (the “biofix” date, understood to represent a reference point in annual *F* *. fusca* development when the rate of the thrips’ dispersal from summer hosts to winter hosts is at its peak during the fall) for each of North Carolina’s climate regions [40]. The rationale for this adjustment is based on the fact that winter conditions are observed first in the mountainous west of North Carolina, later in the piedmont region of the state, and lastly in the coastal plain. Senescence of annual plants causes thrips dispersal from these plants in the flight peak associated with biofix, and this senescence is induced by winter temperatures and day lengths. For the models of Morsello and Kennedy, this biofix date is November 1; for our purposes it is October 21 for counties in the cooler eastern mountains region of the state, November 1 for counties in the piedmont region, and November 7 for counties in the warmer coastal plain region. This adjustment slightly increases the accuracy of thrips dispersal estimates across the state (data not shown).

We treated two estimates of dispersing thrips in our analysis: one from the spring immediately preceding the summer for which TSWV incidence is reported in tobacco, and one from the spring one year before the incidence report (e.g., for reported incidence in tobacco for summer of 2009, this would be the spring of 2008). We refer to the second of these estimates as “prior year thrips,” and expect it to capture an important aspect of virus abundance that winter climate will not detect: abundance of TSWV in prior-year summer hosts, which will affect initial abundance in the winter hosts from which thrips transmit TSWV to tobacco in the spring. After winter hosts become infected with TSWV, subsequent spread in these hosts is determined by thrips population growth and dispersal, affected by winter temperatures and precipitation [5]. Thus prior year thrips numbers are expected to correlate with the amount of virus available for spread into tobacco in a given spring.

Transmission of TSWV to winter hosts in the fall occurs when thrips disperse from their summer hosts, primarily summer annual weeds. Transmission from summer to winter hosts in the fall corresponds to a period of thrips activity during which relatively few thrips are briefly active before populations overwinter [29]. Small numbers of thrips, brevity of the period of transmission, and year-to-year consistency of this fall transmission event mean that the trajectory established during the preceding spring is more determinant of virus abundance in winter hosts than is the relatively small amount of variation in transmission dynamics during the fall.

### Model development

TSWV incidence models for tobacco were assembled using informative weather variables from two general groups: first, those expected to relate primarily to virus abundance in the landscape before transmission to cultivated tobacco; and second, those expected to relate primarily to the intensity of transmission to tobacco. Virus abundance descriptors (e.g. winter temperatures) were chosen based on our understanding of weather’s effects on the plants that constitute TSWV’s winter host reservoir [30,31], and based on our understanding of weather’s effects on the action of overwintering thrips in moving TSWV between these plants [5,6]. Transmission intensity descriptors (e.g. estimated numbers of dispersing thrips) were chosen based on our understanding of weather’s effects on thrips activity and its effects on TSWV transmission, and included average monthly temperature and precipitation variables from spring months.

Because the disease-causing virus in this system is found each year in winter annual plants before being transmitted to tobacco by dispersing thrips [30], we considered weather from October through March as the weather with relevance to these annuals in terms of their role in the TSWV-tobacco disease system. We constrained the potential combinations of descriptors in disease incidence models such that it was necessary for them to include at least one potential description of virus abundance and at least one description of transmission intensity based on weather. Expecting an interaction between an effect describing virus abundance and an effect describing transmission intensity to be significant, we proposed this as interactions between virus- and transmission-related descriptors in potential models.

We wished to make inference about weather descriptors and the thrips estimates derived from them, while treating county-level variation arising from NCCES agents’ assessment practices as random variation, leading us to a generalized linear mixed model framework. Parameter estimation for models of disease incidence based on virus abundance and transmission intensity was conducted using the GLIMMIX procedure of the SAS System [41]. A beta response distribution with a logit link function was used for fitting, and parameter estimation was by Laplace’s integral approximation method to allow comparison of models with different fixed effects by means of Akiake’s Information Criterion [42]. Reported application of imidacloprid (a pesticide commonly applied in tobacco cultivation as a disease-suppressive and insect control measure) was used to adjust reported TSWV incidence. Based on experimental evidence that imidacloprid application decreases TSWV incidence by a factor of 0.3 to 0.5 in field populations of tobacco [36–39], incidence reports for acreage treated with imidacloprid were multiplied by 1.429, representing an estimate of TSWV suppression (decrease by a factor of 0.3) at the conservative end of the range.

### Model selection

A best-fit model was chosen based on minimizing the value of Akiake’s information criterion (AIC), for further scrutiny relative to similarly-parameterized models. This best-fit model was compared to other models each including only a subset of the fixed effects in the best-fit model, to assess whether significant differences exist between the best-fit and alternative models, on the basis of likelihood-ratio tests comparing the relevant pairs of models as nested HYPOTHESIS 10-fold cross-validation was conducted to test the stability of the best-fit model.

## Results

### Test for difference between NCCES and field survey datasets

The paired t-test for difference between NCCES and our field survey datasets detected no significant difference (Student’s *t*; p = 0.49).

### TSWV incidence in Tobacco

Models, listed by included parameters, and corresponding AIC values are shown in Table 1. Each less-parameterized model shown had a significantly poorer fit than the best-fit model, assessed by likelihood-ratio tests. Each more-parameterized model shown did not have a significantly greater fit than the best-fit model, also assessed by likelihood-ratio tests. We tested additional combinations of parameters in models that were not able to be treated as a hypothesis nested within the best-fit model, and in which the best-fit model could not be treated as a nested hypothesis; none of these models had an AIC value less than that of the best-fit model, and examples are shown in Table 1.

**Table 1:** Listing of Akiake’s Information Criterion values (column label AIC) for models described by their included fixed effects.

**Included Fixed Effects**	**AIC**	***LRD***
**PYT MP AWT PYTxMP**	*-352.38*	n/a
PYT MP AWT PYTxMP *AWTxMP*	-350.71	0.33
PYT MP AWT PYTxMP *PYTxAWT*	-350.42	0.04
PYT MP AWT PYTxMP *TT*	-350.41	0.03
MP AWT	-349.26	7.12**
PYT MP AWT	-347.54	6.84**
PYT MP AWT *PYTxTT*	-346.33	n/a
PYT MP PYTxMP	-345.73	8.65**
PYT MP AWT *TT*	-345.65	n/a

All AIC values are reported for generalized linear mixed models in which only the listed fixed effects appear, and in which the County variable is treated as a random effect. Abbreviations for fixed effects are as follows: PYT = prior-year thrips estimate (in units of dispersing adult thrips caught on sticky traps); MP = March precipitation in centimeters; AWT = average winter temperature in degrees Celsius; TT = current-year thrips estimate (in same units as PYT). Thrips estimates are for cumulative adult dispersal from April 1 through May 31. The best-fit model appears in the first row, in bold. Where fixed effects are added to models relative to the best-fit model, they are italicized. The likelihood-ratio *D* statistic is reported (column label LR D) where models can be compared with the best-fit model as nested hypotheses, and asterisks indicate significance of the likelihood-ratio test at the 0.05 (*) and <0.005 (**) level. Where models cannot be compared with the best-fit model as nested hypotheses, “n/a” appears in the LR D column.

The best-fit model chosen by minimum-AIC relates the linear predictor η:

η = 0.0219(PYT) + 0.0916(MP) + 0.156(AWT) - 0.00282(PYT × MP) + *Z*(Co) -5.369

to TSWV incidence through the inverse logit link function

*e* ^η^(1+*e* ^η^)^-1^ = TSWV

where TSWV is disease incidence as a county-wise proportion of infected plants in the absence of imidacloprid treatment, PYT is the prior-year thrips estimation, MP is cumulative March precipitation in centimeters, AWT is average winter temperature in degrees Celsius, and Z is the random effect coefficient corresponding to the given county, Co.

**Figure 3:**
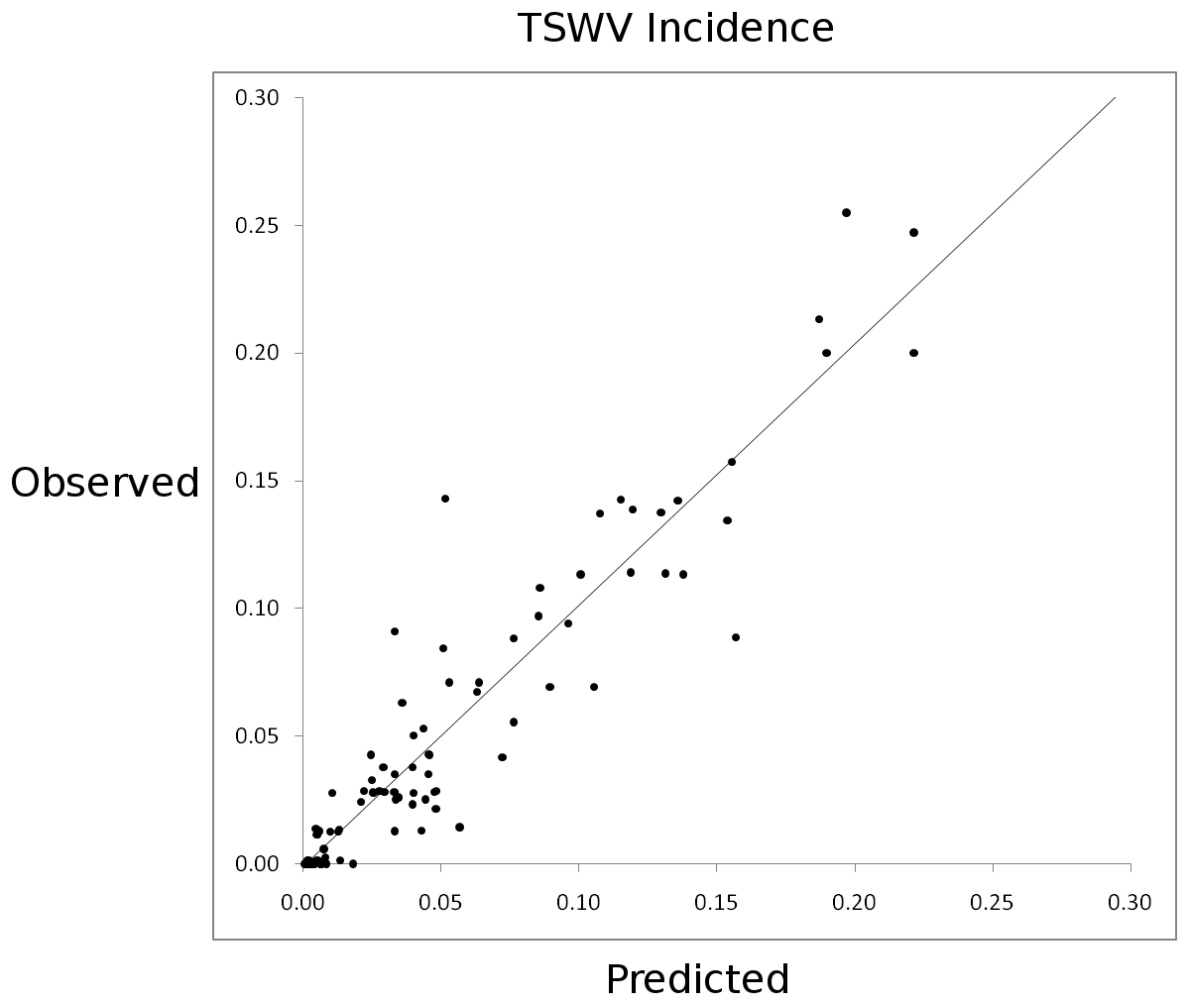
Prediction plot for best-fit model, a regression of observed on fitted TSWV incidence, R^2^ = 0.899.

**Figure 4:**
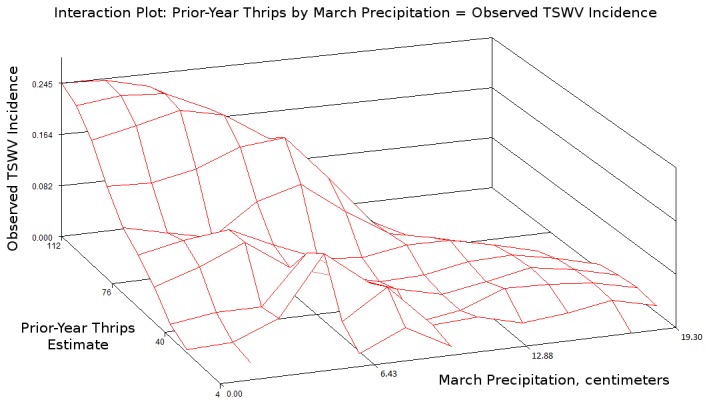
Interaction effect in observed data, showing change in influence of prior-year thrips variable across range of March precipitation, and to lesser extent, *vice versa*.

**Figure 5:**
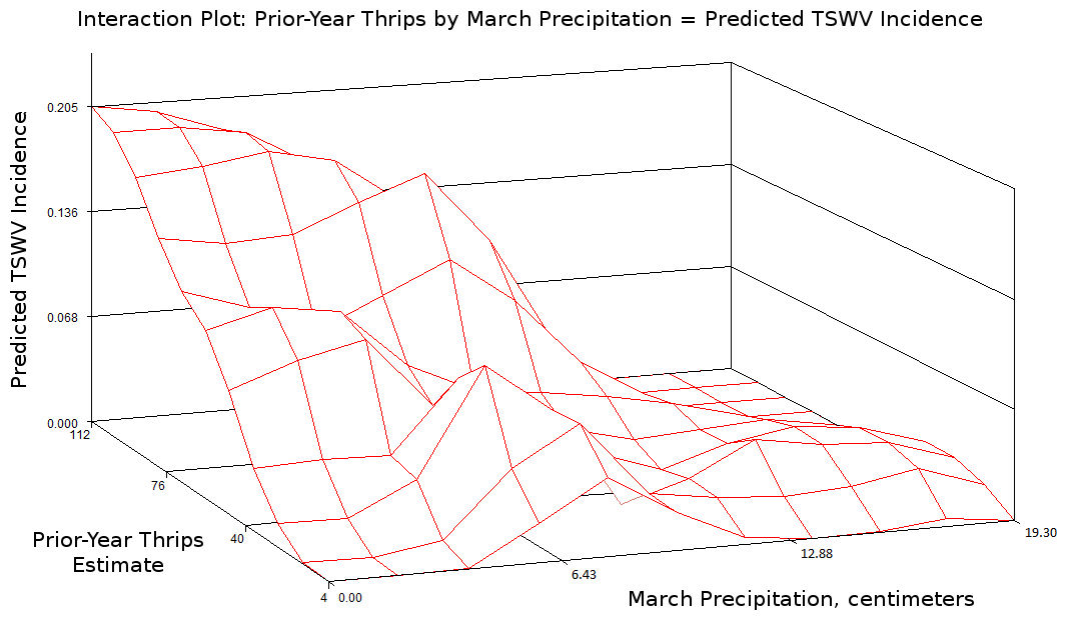
Interaction effect in fitted data, demonstrating that the best-fit model accounts for the interaction.

**Figure 6:**
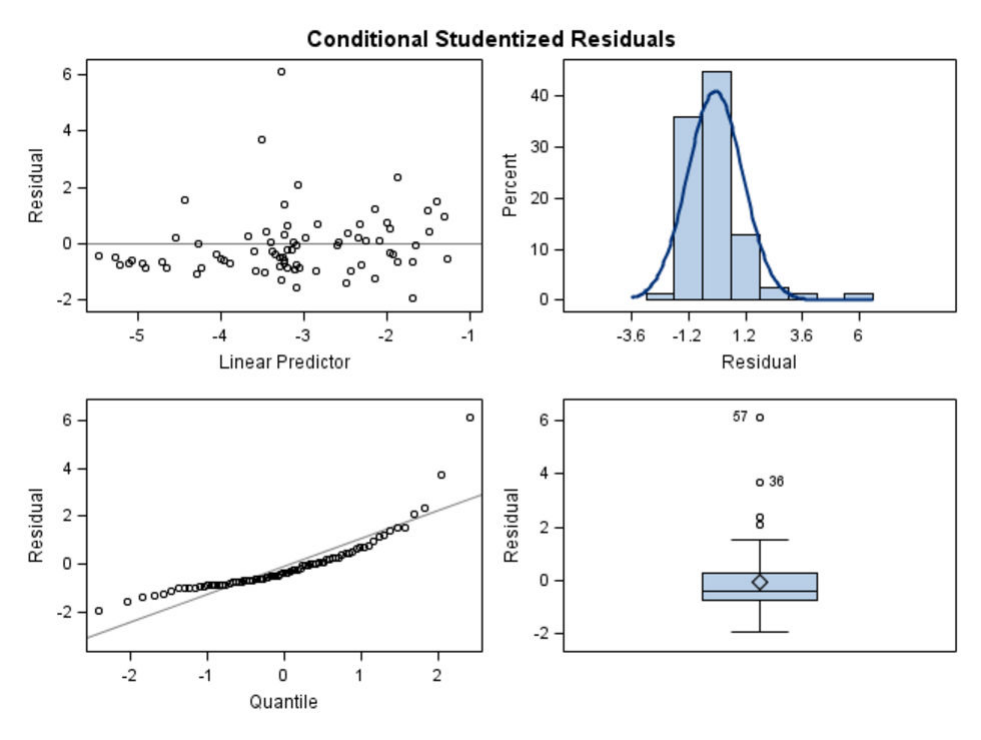
Panel of diagnostic plots for best-fit model, showing conditional Studentized residuals.

The regression of observed on fitted TSWV incidence for this model is shown in Figure 3. A significant interaction between prior-year thrips and March precipitation was found (Figure 4), and is accurately captured by the best-fit model (Figure 5). A description of conditional Studentized residuals for this model is shown as a panel in Figure 6.

Cross-validation confirmed stability of prediction error (SD of RMSE for all folds = 0.008, average RMSE = .0273). For each of 10 folds, the model constructed using the training set was significant in describing the validation set (*p*<0.05). Averages and standard deviations for parameter estimates from the ten cross-validation models are shown in Table 2.

**Table 2:** Listing of coefficient averages and standard deviations (SD) from a 10-fold cross-validation of the best-fit model.

**Parameter**	**Average**	**SD**	**Best-fit Estimate**
**Intercept**	-5.353	0.132	-5.369
**PYT**	0.0224	0.00297	0.0219
**MP**	0.0886	0.00923	0.0916
**AWT**	0.156	0.0208	0.156
**PYT x MP**	-0.00285	0.000319	-0.00282

Parameter estimates from the best-fit model (trained on the entire dataset) are shown for comparison.

## Discussion

We studied the relationship of weather to TSWV incidence in tobacco, recognizing a degree of independence between each of two groups of components mediating the effects of weather: virus abundance in the plants constituting the natural reservoir of TSWV, and transmission intensity mediated by the activity of tobacco thrips. This recognition led to treating the effects of weather differently for virus abundance *vs.* transmission intensity and uncovered a strong interaction between them in determining TSWV incidence. We interpret the relationship between the fixed effects that compose the best-fit model and the observation of TSWV incidence in tobacco as follows. Prior-year thrips abundance constitutes an effect that is positively correlated with virus abundance during the current year’s spring, as prior-year transmission intensity establishes the abundance of virus in summer, and subsequently, winter annual weeds. Average winter temperature affects the abundance and persistence of winter annual weeds, as well as the action of thrips in spreading infection between these weeds, such that warmer temperatures lead to longer overlap between summer and winter annuals during the fall, and more abundant winter annuals during the winter – both reasonably expected to correlate positively with virus abundance in the landscape during the ensuing spring. March precipitation is known to have a suppressive effect on dispersal activity of adult thrips, and to have a lingering effect on thrips populations through inducing mortality to juveniles [6], and is thus understood to correlate negatively with transmission intensity. The interaction between prior-year thrips and March precipitation realizes the conditionality of disease incidence on these interacting factors.

We interpret the interaction between prior-year thrips and March precipitation as a description of the relationship between virus abundance and transmission intensity. Warmer winters are associated with larger populations of thrips, as well as greater abundance of winter host plants among which thrips transmit TSWV. Spread of TSWV among winter hosts by thrips during a warm winter leads to high availability of virus for transmission by thrips in the spring. Transmission during the spring is strongly influenced by precipitation, especially during the month of March. Virus abundance and transmission intensity each influence disease incidence, but conditionally based on the other factor. High virus abundance leads to high disease incidence, provided transmission is intense; and high transmission intensity leads to high disease incidence, provided virus is abundant.

In many years, temperature averages and precipitation totals for cold-season months and warm-season months are correlated, but deviations from this pattern in North Carolina are not uncommon (e.g., 2002/2003, 2010/2011), potentially due to ENSO patterns. We find that a climatic transition from a relatively warm winter to a relatively cool spring represents an appreciable effect on disease dynamics in this system, as do transitions from relative cool to warm temperatures, or similar transitions concerning precipitation. The 2002/2003 and 2010/2011 transitions were each characterized by a relatively warm winter (generally thrips-favorable and supportive of natural hosts of TSWV, and therefore conducive to increased virus abundance) followed by a relatively cool and rainy spring (with a generally negative effect on thrips population growth, and suppressive effect on thrips vectoring activity [6]). Both transitions corresponded to changes in ENSO state, specifically changes from EN/LN to ENSO neutrality. And in both cases, winter and spring climates’ roles in affecting TSWV risk are evidently interdependent, reflecting the biological basis of TSWV incidence: a requirement for both weather supporting high virus abundance, and weather supporting thrips-mediated transmission, in order for disease to materialize.

In the currency of predictive value, separating virus abundance and thrips activity to some degree adds value by mitigating the possibility of a severe over- or under-estimation of incidence. This is because such treatment explicitly recognizes that both factors are required for disease incidence to be realized. Disease incidence may be correlated with a metric that relates to one or another component of the disease system as a whole because other components of that disease system are typically correlated in a similar way; however, in cases where conditions result in these components’ not being coordinated (as we find in the case of TSWV in North Carolina during 2002-2003), an estimate based on one component but ignoring the compensatory or interacting effect of another will fail to give a realistic incidence estimate. Our results corroborate this by demonstrating a significant effect of a two-way interaction between descriptors that attempt to approximate virus abundance and thrips activity for statistical analysis. Recognizing the importance of both virus and vector in a model of incidence also offers a greatly improved description of incidence, *vs.* existing model frameworks that focus on factors that affect virus abundance, e.g. [13]. We argue that accuracy in risk estimation under unusual conditions – those associated with the highest and lowest disease incidence observations – is of primary importance to a model aimed at describing an infectious diseases system wherein epidemics and crashes are expected. In this system, such events are found to be brought about by conditions favorable or antagonistic to the spring transmission of TSWV by thrips.

In this study, we have built on the understanding of disease dynamics in agroecosystems, and on work describing the relationship of weather to the activity of a vector, by placing vector activity into the greater context of an annual disease system cycle. We conclude that conditions far in advance of a given cropping season can influence disease incidence, indicating the importance of understanding the biology of TSWV in its natural hosts throughout the year. Because thrips-mediated transmission affects TSWV disease dynamics in winter annuals, clarifying their role in the agricultural disease system will enhance our ability to understand epidemiological aspects of disease in crops. More broadly, clarifying the interface of virus abundance and transmission intensity by better characterizing their multiple and different dependencies on factors such as weather, as well as larger-scale climatological factors, will enhance our understanding of plant disease epidemiology in general.

## References

[1] Nault LR (1997) Arthropod Transmission of Plant Viruses: A New Synthesis. Ann Entomol Soc Am 90: 521-541.

[2] Spence NJ (2001) Virus-vector interactions in plant virus disease transmission and epidemiology. In: Jeger MJ Spence NJ Biotic Interactions in Plant-Pathogen Interactions. Wallingford, UK: CAB International pp. 15–26.

[3] Madden LV, Raccah B, Pirone TP (1990) Modeling plant disease increase as a function of vector numbers: non persistent viruses. Researches Popul Ecol 32: 47–65. doi:10.1007/BF02512589.

[4] Madden LV, Knoke JK, Louie R (1983) The statistical relationship between aphid trap catches and maize dwarf mosaic virus inoculation pressure. In: Plumb RT Thresh JM Oxford: Plant Virus Epidemiology: Blackwell. pp. 159–168

[5] Morsello SC, Kennedy GG (2009) Spring temperature and precipitation affect tobacco thrips, *Frankliniella fusca* (Thysanoptera: Thripidae) population growth and Tomato spotted wilt virus within patches of the winter weed *Stellaria media* . Entomol Exp Applicata 130: 138-148. doi:10.1111/j.1570-7458.2008.00801.x.

[6] Morsello SC, Beaudoin ALP, Groves RL, Nault BA, Kennedy GG (2010) The influence of temperature and precipitation on spring dispersal of *Frankliniella fusca* changes as the season progresses. Entomol Exp Applicata: 134-260.

[7] Jeger MJ, van den Bosch F, Madden LV, Holt J (1998) A model for analysing plant–virus transmission characteristics and epidemic development. Math Med Biol 15: 1-18. doi:10.1093/imammb/15.1.1.

[8] Madden LV, Jeger MJ, van den Bosch F (2000) A Theoretical Assessment of the Effects of Vector-Virus Transmission Mechanism on Plant Virus Disease Epidemics. Phytopathology 90: 576-594. doi:10.1094/PHYTO.2000.90.6.576. PubMed: 18944537. 18944537 10.1094/PHYTO.2000.90.6.576

[9] Ullman DE, Meideros R, Campbell LR, Whitfield AE, Sherwood JL et al. (2002) Thrips as vectors of tospoviruses. Adv Bot Res 36: 113-140. doi:10.1016/S0065-2296(02)36061-0.

[10] Stinner RE, Gutierrez AP, Butler GD Jr. (1974) An algorithm for temperature-dependent growth rate simulation. Can Entomol 106: 519-524. doi:10.4039/Ent106519-5.

[11] Getz WM, Gutierrez AP (1982) A perspective on systems analysis in crop production and insect pest management. Annu Rev Entomol 27: 447-466. doi:10.1146/annurev.en.27.010182.002311.

[12] Holt J, Jeger MJ, Thresh JM, Otim-Nape GW (1997) An Epidemiological Model Incorporating Vector Population Dynamics Applied to African Cassava Mosaic Virus Disease. J Appl Ecol 34: 793-806. doi:10.2307/2404924.

[13] Mila AL (2011) Explaining Loss Caused by Tomato spotted wilt virus on Tobacco with Boreal Winter Weather: A Bayesian Approach. Phytopathology 101: 462-469. doi:10.1094/PHYTO-05-10-0146. PubMed: 21091184. 21091184 10.1094/PHYTO-05-10-0146

[14] Coutts BA, Thomas-Carroll ML, Jones RAC (2004) Patterns of spread of Tomato spotted wilt virus in field crops of lettuce and pepper: spatial dynamics and validation of control measures. Ann Appl Biol 145: 231-245. doi:10.1111/j.1744-7348.2004.tb00379.x.

[15] Coutts BA, Jones RAC (2005) Suppressing spread of Tomato spotted wilt virus by drenching infected source or healthy recipient plants with neonicatinoid insecticides to control thrips vectors. Ann Appl Biol 146: 95-103. doi:10.1111/j.1744-7348.2005.04033.x.

[16] Momol MT, Olson SM, Funderburk JE, Stavisky J (2004) Integrated management of tomato spotted wilt on field-grown tomatoes. Plant Dis 88: 882-890. doi:10.1094/PDIS.2004.88.8.882. 10.1094/PDIS.2004.88.8.882 30812519

[17] Reitz SR, Yearby EL, Funderburk JE, Stavisky J, Momol ML et al. (2003) Integrated management tactics for *Frankliniella* thrips (Thysanoptera: Thripidae) in field-grown peppers. J Econ Entomol 96: 1201-1214. doi:10.1603/0022-0493-96.4.1201. PubMed: 14503592. 14503592 10.1603/0022-0493-96.4.1201

[18] Riley DG, Pappu HR (2004) Tactics for management of thrips (Thysanoptera: Thripidae) and Tomato spotted wilt virus in tomato. J Econ Entomol 97: 1648-1658. doi:10.1603/0022-0493-97.5.1648. PubMed: 15568355. 15568355 10.1603/0022-0493-97.5.1648

[19] Hagan AK, Weeks JR, French C, Gudauskas RT, Mullen JM et al. (1990) Tomato spotted wilt virus in peanut in Alabama. Plant Dis 74: 615. doi:10.1094/PD-74-0615E.

[20] Chamberlin JR, Todd JW, Beshear RJ, Culbreath AK, Demski JW (1992) Overwintering Hosts and Wingfonn of Thrips, *Frankliniella* spp., in Georgia (Thysanoptera: Thripidae): Implications for Management of Spotted Wilt Disease. Environmental Entomology 21: 121-128.

[21] Gitaitis RD, Dowler CC, Chalfant RB (1998) Epidemiology of tomato spotted wilt in pepper and tomato in southern Georgia. Plant Dis 82: 752-756. doi:10.1094/PDIS.1998.82.7.752. 10.1094/PDIS.1998.82.7.752 30856944

[22] Pappu SS, Pappu HR, Gitaitis RD, Gay JD (1998) First report of tomato spotted wilt Tospovirus infection of watermelon in Georgia. Plant Dis 82: 351-351. doi:10.1094/PDIS.1998.82.3.351C. 10.1094/PDIS.1998.82.3.351C 30856877

[23] Chatzivassiliou EK (2008) Management of the Spread of Tomato spotted wilt virus in Tobacco Crops with Insecticides Based on Estimates of Thrips Infestation and Virus Incidence. Plant Dis 92: 1012-1020. doi:10.1094/PDIS-92-7-1012. 10.1094/PDIS-92-7-1012 30769537

[24] Groves RL, Sorenson CE, Walgenbach JF, Kennedy GG (2001) Effects of imidacloprid on transmission of tomato spotted wilt Tospovirus to pepper, tomato and tobacco by *Frankliniella fusca* Hinds (Thysanoptera: Thripidae). Crop Protect 20: 439-445. doi:10.1016/S0261-2194(00)00171-X.

[25] Csinos AS, Pappu HR, McPherson RM, Stephenson MG (2001) Management of Tomato spotted wilt virus in flue-cured tobacco with acibenzolar-S-methyl and imidacloprid. Plant Dis 85: 292–296. doi:10.1094/PDIS.2001.85.3.292. 10.1094/PDIS.2001.85.3.292 30832045

[26] Kirk WDJ (1997) Distribution, abundance and population dynamics. In: Lewis T Thrips as Crop Pests. Oxon, UK: CAB International pp. 217–257.

[27] Mila AL, Radcliff J (2009) Managing diseases. In: Flue-Cured Tobacco Production Guide. Raleigh, NC: Cooperative Extension Service, North Carolina State University . pp. 140-174

[28] McPhaden MJ (2003) Evolution of the 2002/03 El Niño. Bulletin American Meteorological Society 85: 677–695.

[29] Groves RL, Walgenbach JF, Moyer JW, Kennedy GG (2003) Seasonal dispersal patterns of *Frankliniella fusca* (Thysanoptera: Thripidae) and *Tomato spotted wilt virus* occurrence in central and eastern North Carolina. J Econ Entomol 96: 1-11. doi:10.1603/0022-0493-96.1.1. PubMed: 12650337. 12650337 10.1603/0022-0493-96.1.1

[30] Groves RL, Walgenbach JF, Moyer JW, Kennedy GG (2001) Overwintering of *Frankliniella fusca* (Thysanoptera: Thripidae) on winter annual weeds infected with *Tomato spotted wilt virus* and patterns of virus movement between susceptible weed hosts. Phytopathology 91: 891-899. doi:10.1094/PHYTO.2001.91.9.891. PubMed: 18944235. 18944235 10.1094/PHYTO.2001.91.9.891

[31] Groves RL, Walgenbach JF, Moyer JW, Kennedy GG (2002) The role of weed hosts and tobacco thrips, *Frankliniella fusca*, in the epidemiology of *Tomato spotted wilt virus* . Plant Dis 86: 573-582. doi:10.1094/PDIS.2002.86.6.573. 10.1094/PDIS.2002.86.6.573 30823226

[32] Kahn ND, Walgenbach JF, Kennedy GG (2005) Summer weeds as hosts for *Frankliniella occidentalis* and *Frankliniella fusca* (Thysanoptera: Thripidae) and as reservoirs for *Tomato spotted wilt virus* . J Econ Entomol 98: 1810-1815. doi:10.1603/0022-0493-98.6.1810. PubMed: 16539098. 16539098 10.1093/jee/98.6.1810

[33] Hobbs HA, Black LL, Story RN, Valverde RA, Bond WP et al. (1993) Transmission of *Tomato spotted wilt virus* from pepper and three weed hosts by *Frankliniella fusca* . Plant Dis 77: 797–799. doi:10.1094/PD-77-0797.

[34] Lowry VK, Smith JW, Mitchell FL (1992) Life-Fertility Tables for *Frankliniella fusca* (Hinds) and *F. occidentalis* (Pergande) (Thysanoptera: Thripidae) on Peanut. Ann Entomol Soc Am 85: 744-754.

[35] Ullman DE, Cho JJ, Mau RFL, Hunter WB, Westcot DM et al. (1992) Thrips-tomato spotted wilt virus interactions: morphological, behavioral and cellular components inﬂuencing thrips transmission. In: Harris KF Advances in Disease Vector Research: v. 9 New York: Springer-Verlag pp. 195–240.

[36] Pappu HR, Csinos AS, McPherson RM, Jones DC, Stephenson MG (2000) Effect of acibenzolar-S-methyl and imidacloprid on suppression of tomato spotted wilt Tospovirus in flue-cured tobacco. Crop Protect 19: 349–354. doi:10.1016/S0261-2194(00)00028-4.

[37] Groves RL, Sorenson CE, Walgenbach JF, Kennedy GG (2001) Effects of imidacloprid on transmission of tomato spotted wilt Tospovirus to pepper, tomato and tobacco by *Frankliniella fusca* Hinds (Thysanoptera: Thripidae). Crop Protect 20: 439-445. doi:10.1016/S0261-2194(00)00171-X.

[38] Melton TA, Fountain C, Gutierrez W, Broadwell A, Wilson J (2004) Chemical control of tomato spotted wilt in flue-cured tobacco. In: Flue-cured tobacco.

[39] Cherry K, Mila A, Fountain C, Radcliff J (2009) Control of tomato spotted wilt in flue-cured tobacco, 2008 Plant Disease Management Report 3: FC084 doi: 10.1094/PDMR03

[40] http://www.climatechange.nc.gov/pages/climatechange/climate_maps_nc.pdf.

[41] Littell RC, Milliken Ga, Stroup WW, Wolfinger RD (1996) SAS System for Mixed Models. SAS Institute Inc., Cary, NC.

[42] Stroup WW (2012) Generalized Linear Mixed Models: Modern Concepts, Methods and Applications. Boca Raton, FL: CRC Press.

