# Correction: Interacting Virus Abundance and Transmission Intensity Underlie Tomato Spotted Wilt Virus Incidence: An Example Weather-Based Model for Cultivated Tobacco

**DOI:** 10.1371/journal.pone.0197565

**Published:** 2018-05-14

**Authors:** Thomas M. Chappell, Amanda L. P. Beaudoin, George G. Kennedy

The term “Akaike’s Information Criterion” is misspelled throughout the article. The correct term is “Akaike’s Information Criterion.”
